# Mitochondrial genome of *Bactrocera tuberculata u*sing next-generation sequencing from China and its phylogenetic implication

**DOI:** 10.1080/23802359.2020.1775517

**Published:** 2020-06-11

**Authors:** Tao Wang, Yanling Ren, Zhongshan Liu, Quwen Lei, Jian Tang

**Affiliations:** a Guizhou Light Industry Technical College, Guiyang, P. R. China; b Guiyang Customs, Guiyang, P. R. China; c Kunming Customs Technical Center, Kunming, P. R. China

**Keywords:** Mitogenome, Dacinae, *Bactrocera tuberculata*, phylogeny

## Abstract

The complete mitochondrial genome (mitogenome) of *Bactrocera tuberculata* (Diptera: Tephritidae: Dacinae) was sequenced and annotated. The mitochondrial genome is 15,273 bp (GenBank No. MT196006), containing 72.2% A + T (A 38.4%, C 16.9%, G 10.9%, and T 33.8%), which is the classical structure for insect mitogenome. All PCGs were started with ATN (ATA/ATG/ATT/ATC) and terminated with TAA except ND3, which ends with TAG. Additionally, the phylogenetic tree confirmed that *B. tuberculata* was closely related to *Bactrocera diaphora* and *Bactrocera oleae*. The current study would enrich the information about mitogenomes of the fruit flies.

*Bactrocera tuberculata* belongs to *Bactrocera* genus, which was erected by Macquart in 1983. It is an important pest of fruit and its host is peach, mango, etc. It was found in Thailand, Myanmar, Vietnam, Laos, China. The identification of *B. tuberculata* is mainly based on morphology and partial sequence. Presently, we have sequenced and determined the complete mitochondrial genome (mitogenome) using the next-generation sequencing method for the first time, which might facilitate future studies on the subfamily Dacinae.

The genome DNA was extracted from male adult of *B. tuberculata* which was collected in Yonghe port, Lincang city, Yunnan province, China (E 99°14′3″, N 23°5′43″), in August 2019, and the voucher specimen’s genome DNA is deposited in the Research Center for Guizhou Characteristic Fruits and its Products of Mountainous Regions, Guizhou Light Industry Technical College, label number is GCZX-140. These sequences were assembled using Geneious Primer (Kearse et al. 2012), version 10.2.3 (http://www.geneious.com/). Additionally, all tRNAs were found by MITOS server (http://mitos.bioinf.uni-leipzig.de/index.py) (Bernt et al. 2013) and tRNA scan-SE server (Lowe and Chan 2016) was used for annotation. The neighbor-joining (NJ) tree was constructed to investigate the molecular taxonomic position of *B. tuberculata* based on the nucleotide sequences of 13 protein-coding genes and 2 rRNA genes using MEGA 6.0 (Tamura et al. 2013) from alignments created by the MAFFT (Katoh and Standley 2013).

The complete mitogenome of *B. tuberculata* is 15,273 bp (GenBank No. MT196006), containing 22 transfer RNA genes (tRNAs, 1,467 bp), 13protein-coding genes (PCGs, 11,316 bp), 2 ribosomal RNA genes (rRNAs, 2,110 bp), and 1 non-coding region (Control region, 251 bp). The whole genome contained 38.4% A, 16.9% C, 10.9% G, and 33.8%T, showing an obvious A + T bias (72.2%). The AT-skew (0.0637) for the whole mitogenome is slightly positive, but negative for GC-skew (–0.2158). All PCGs started with ATN (ATA/ATG/ATT/ATC) and terminated with TAA except ND3, which ends with TAG.

The phylogenetic relationships of *B. tuberculata* were reconstructed using the neighbor-joining (NJ) method with 1000 bootstrap replicates based on the concatenated nucleotides of the 13 PCGs and 2 rRNAs with 13,426 bp, *Dosophila suzukii and Drosophila melanogaster* were used as outgroup (Figure 1). The phylogenetic tree confirms that *B. tuberculata* was closely related to *B. diaphora* and *B. oleae*. Presently, the studies recording *B. tuberculata* are limited, and we believe that our data could be useful for further study.

**Figure 1.**
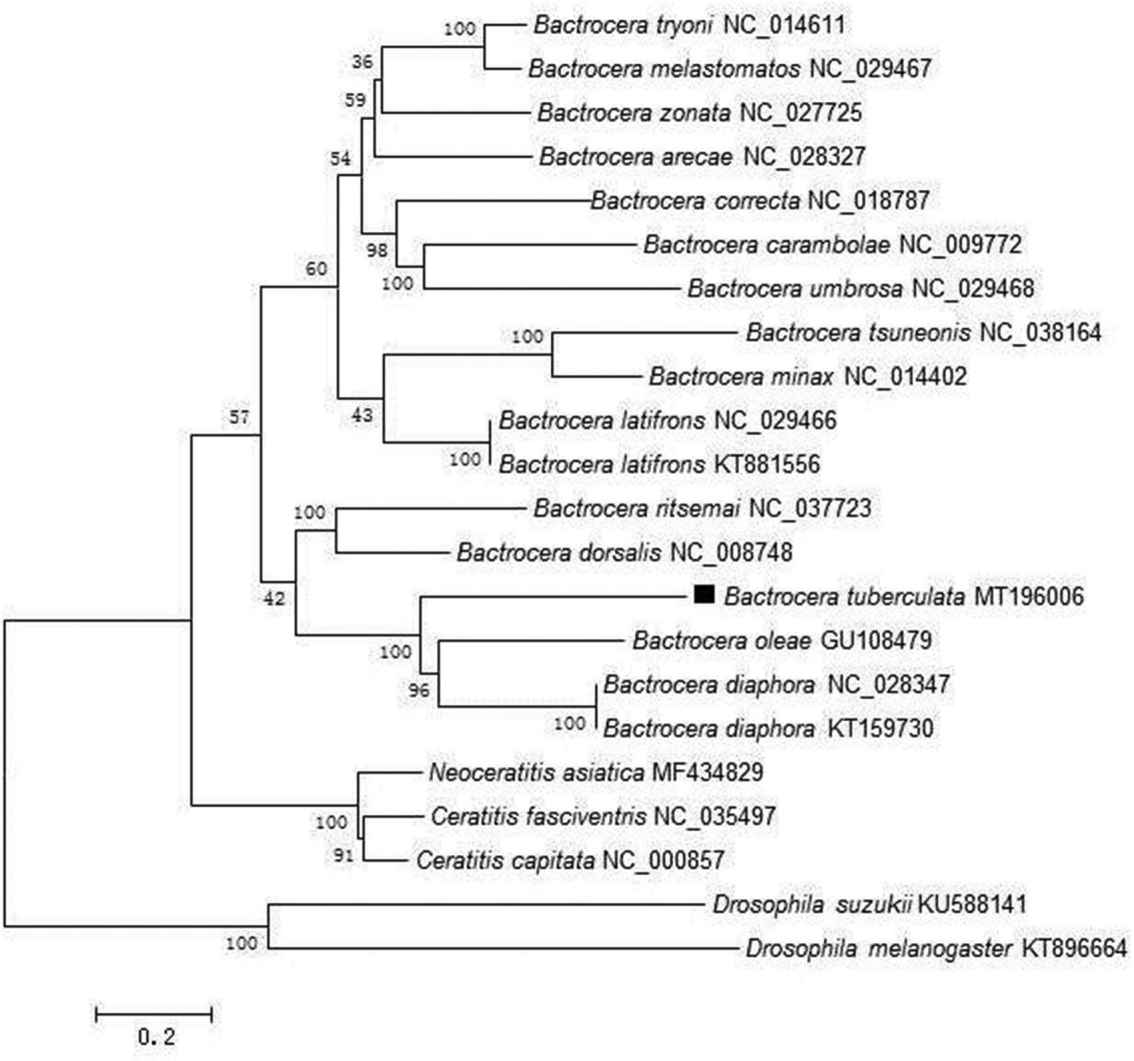
Neighbor-joining (NJ) phylogenetic tree of *Bactrocera tuberculata* based on concatenated nucleotides of the 13 PCGs and 2 rRNAs by MEGA 6.0.

## Data Availability

The data that support the findings of this study are openly available in NCBI at https://www.ncbi.nlm.nih.gov/, reference number MT196006.
